# Factors Affecting Mortality in 1022 COVID-19 Patients Referred to an Emergency Department in Bergamo during the Peak of the Pandemic

**DOI:** 10.1007/s42399-020-00444-4

**Published:** 2020-08-17

**Authors:** Orlando Goletti, Chiara Nessi, Amidio Testa, Giovanni Albano, Valter Torri, Giordano Domenico Beretta, Massimo Castoldi, Emilio Bombardieri

**Affiliations:** 1 General Surgery Unit, Humanitas Gavazzeni, Bergamo, Italy; 2 School of Specialization of General Surgery, University of Verona, Verona, Italy; 3 Internal Medicine Unit, Humanitas Gavazzeni, Bergamo, Italy; 4 Anesthesia and Intensive Care Unit, Humanitas Gavazzeni, Bergamo, Italy; 5 Clinical Research Methodology Laboratory, Oncology Unit, IRCCS, Mario Negri Institute, Milan, Italy; 6 Medical Oncology Unit, Humanitas Gavazzeni, Bergamo, Italy; 7 Medical Direction, Humanitas Gavazzeni, Bergamo, Italy; 8 Scientific Direction, Humanitas Gavazzeni, Bergamo, Italy

**Keywords:** COVID-19 mortality, Risk factors, Severity, Emergency during the peak of infection

## Abstract

The unexpected outbreak of COVID-19 in the area of Bergamo and the general crisis of personnel and devices has been managed as well as possible during the maximum peak of epidemic; Humanitas Gavazzeni Hospital implemented its facilities and organization in order to optimize the treatment of patients. The number of beds in the Intensive Care Unit (ICU) was doubled (from 16 to 33), and more than 220 beds were dedicated to the COVID-19 patients. This paper analyzes the factors affecting mortality in 1022 COVID-19 patients who referred to Humanitas Gavazzeni between February 25 and March 26, 2020. A total of 274 (34.9%) fatal events were registered: 202 among those admitted to the Intensive Care Unit (ICU) and COVID department and 72 among those treated in Acute Admission Unit Level II (AAUl-2) who died before hospital admission. This paper studies 274 dead cases by analyzing patient’s characteristics, physiological and laboratory parameters, symptoms, and the scores of severity of the disease. Patients who had fatal events in the AAUL-2 showed the worst parameters of risk. The most important differences regarded the Apache II score, Glasgow Coma Score (GCS), CRP (C-reactive protein), pH, creatinine, RR (respiratory rate), and asthenia.

## Introduction

The outbreak of COVID-19 in Italy has recently become a public health emergency of international concern. Northern regions were the most affected with 67,931 positive cases and 12,579 deaths in Lombardy [1, 2]. The province of Bergamo was one of the most damaged, with a rapid increase of positive cases in a very short period [3].

Bergamo’s hospitals had to face a tremendous overload of patients in the Emergency Department. The unpredictable influx of patients determined a deep crisis in personnel, beds, and devices. The facilities were not prepared for such a dramatic event. Humanitas Gavazzeni gave its maximum effort in order to assure the best assistance by implementing the ICU beds, by transforming the normal wards in COVID 19 structures, and by organizing new protocols according to the international WHO recommendations for the pandemic management considering of a lack of validated protocols of specific treatment. It should also be considered that it was not possible to transfer any acute patients to other hospitals due to the spread of the same crises in the other structures of the healthcare system.

A total of 1022 COVID positive patients referred to the Emergency Department: 714 were admitted into the hospital (COVID department and ICU) with respiratory disease and a wide spectrum of clinical manifestations, 236 were discharged at home with therapy prescriptions and daily follow-up, and 72 were observed and treated in a section of the Emergency Department (Acute Admission Unit Level II, AAUL-2) and died before admission.

Within the period of the maximum peak of endemic, 274 fatal cases were registered: 202 hospitalized patients and 72 patients accepted in AAUL-2.

This paper analyzes retrospectively the characteristics of deceased patients and describes the different distributions of parameters of severity of disease between two groups (those dead inside the hospital and those in AAUL-2).

## Methods

We retrospectively evaluated the records of all patients referred to Humanitas Gavazzeni between February 25 and March 26, 2020, during the peak of pandemic. In that period, overall 1812 patients referred to the Emergency Department with different diseases, including COVID pathology. A team of experienced intensivists and anesthesiologists detected 1022 COVID-19 positive patients. Their clinical status was evaluated according to the severity of disease, oxygen desaturation, fever, respiratory symptoms, radiologic imaging and paying attention to several indices of risk [4–7].

The admitted cases with clinical diagnosis of COVID-19 were confirmed by radiology and/or laboratory tests (nasopharyngeal swab). All patients showed severe disease, mainly characterized by persistent fever (> 39 °C), recent worsening dyspnea, cough, and various flu-like symptoms.

The aim of this study was the evaluation of fatal events registered within the considered period. Overall 274 patients died: 202 patients into the COVID-19 department and ICU and 72 in AAUL-2.

We analyzed the data registered at patient presentation: age and sex, physiological and laboratory parameters, symptoms, and presence of comorbidities (cardiovascular diseases, respiratory diseases, arterial hypertension, diabetes, oncological diseases, chronic renal failure, neurological diseases, smoking, and others). We calculated also the Chronic Health Evaluation II (Apache II) [8] score and the Glasgow Coma Score (GCS), as indicators of risk of death, considering the measures obtained within 24 h from the patients’ admission. The informed consent for the scientific utilization and publications of data related to the disease was obtained by the patients at their admission according to the Rules of Humanitas Gavazzeni COVID-19 Emergency Department. This retrospective study was notified to the ICH Ethical Committee (Rozzano-Milano).

## Statistical Methods

Basic demographic characteristics were described by common statistical summary measures (frequencies and proportion for categorical data, mean and standard deviation for continuous variables).

The association between these characteristics and the site of hospitalization, assessed by univariate and multivariate logistic model, were measured with the odds ratio (OR) and 95% CI for each factor.

## Results

The reported analysis did not consider the type of treatments administered to the patients, because of the various changes that occurred over the time. In fact, the therapy of COVID19 patients has undergone revisions in a very short time, due to the subsequent indications and experiences in the national and international centers.

**Fig. 1:**
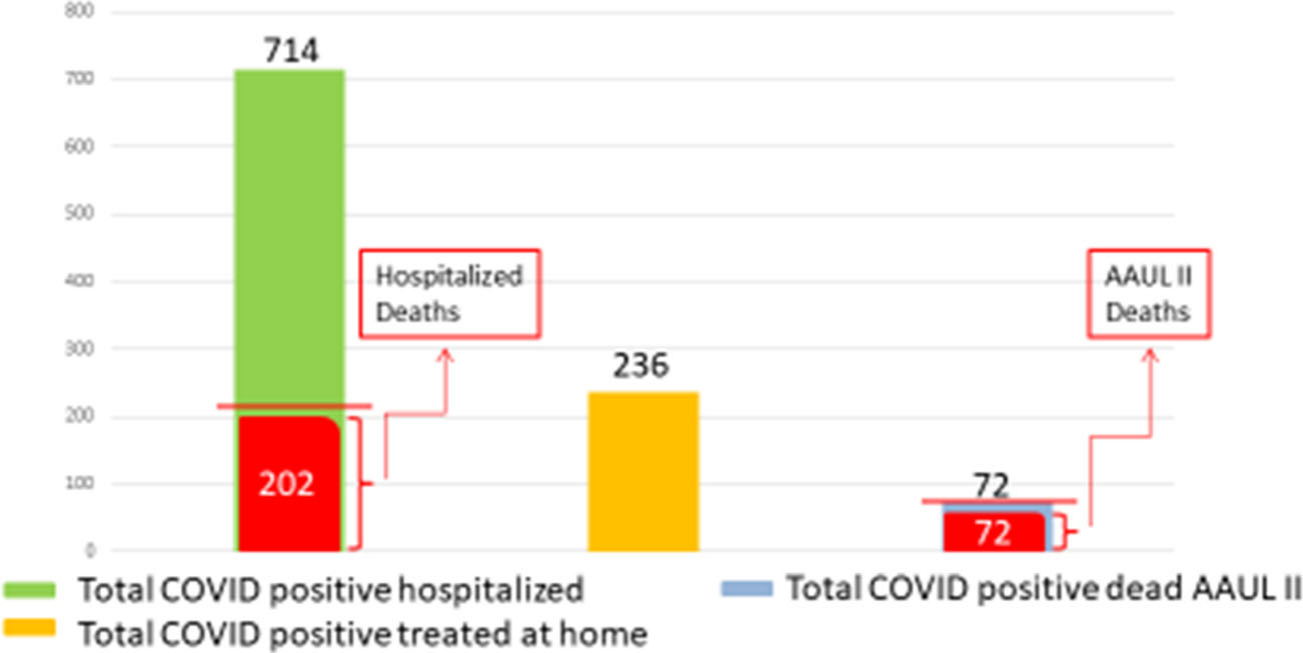
The graph shows 1022 COVID positive patients admitted to the Emergency Department during the peak of the pandemic (25 February to 26 March 2020), divided into those who were hospitalized in the COVID department and ICU (green), those who were discharged with home treatment and daily follow-ups (orange), and those who died in AAUL-2 before the hospitalization (light blue)

All patients who died (72) in AAUL-2 received oxygen therapy and current supportive care, while hospitalized patients (714) received different combinations of treatments according to the protocols discussed within the internal steering committee for COVID-19. In addition, 236 patients were discharged with oxygen desaturation of ≥ 92% and treated at home with daily follow-up (Fig. 1).

**Fig. 2:**
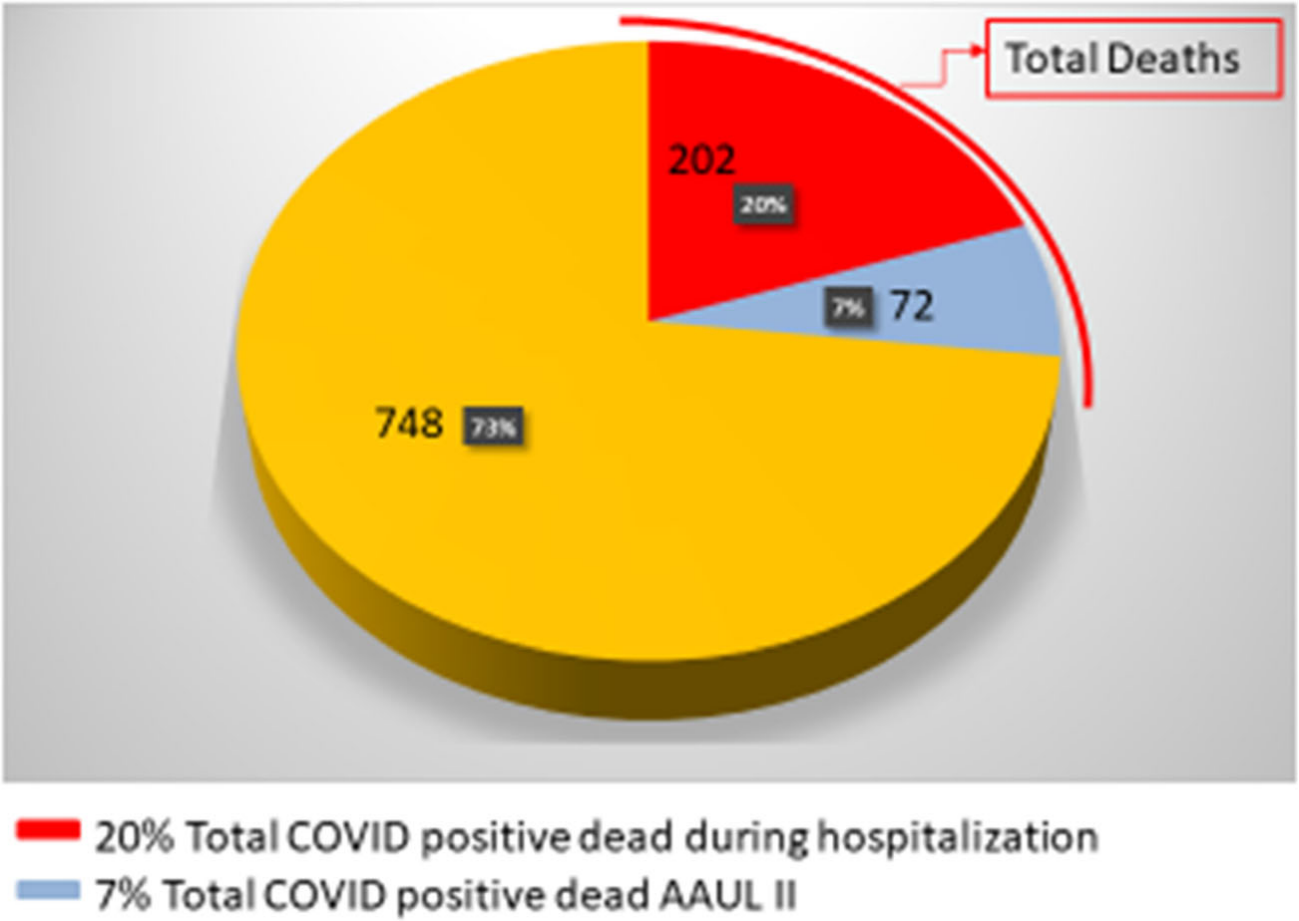
The graph shows 1022 (100%) COVID positive patients admitted during the peak of the pandemic (25 February to 26 March 2020), divided into those who died in the COVID department and ICU (red), those who died in AAUL-2 (light blue), and those who survived (orange)

We analyzed the distribution of the most important physiological and laboratory parameters in the dead patients (274), split into those who died in AAUL-2 (72) and those who died inside the hospital (202). Mean, CI (95%), and *p* value have been reported (Table 1, Fig. 2).

**Table 1:** Patients characteristics at baseline, sign, and symptoms

	Groups of patients						
	AAUL-2 (*N* = 72)		COVID department ICU (*N* = 202)		TOTAL (*N* = 274)		*p* Value for association logistic model
	Mean	Std.	Mean	Std.	Mean	Std.	
AGE	81.6	7.5	77.1	8.4	78.3	8.4	0.899
Apache score	21.0	6.7	14.2	4.2	16.0	5.8	< 0.001
GCS	11.1	4.3	14.6	1.0	13.7	2.8	< 0.001
T	37.6	0.8	37.9	0.9	37.8	0.9	0.017
PAM	83.0	19.4	93.1	16.7	90.5	18.0	< 0.001
Pulse rate	95.6	21.3	92.8	18.7	93.5	19.4	0.283
Resp. rate	24.0	6.9	21.1	4.7	21.9	5.5	< 0.001
Creatininemia	2.1	1.5	1.5	1.0	1.7	1.2	< 0.001
Sodiemia	137.2	7.5	135.7	5.6	136.1	6.2	0.068
Kaliemia	4.1	0.7	4.0	0.6	4.0	0.7	0.313
GB	9.9	5.2	8.2	4.0	8.6	4.4	0.006
HCT	38.8	5.7	39.2	5.4	39.1	5.5	0.606
PCR	23.4	10.7	17.4	9.1	18.9	9.8	< 0.001
pH	7.4	0.1	7.5	0.0	7.5	0.1	< 0.001
pO2	45.4	19.5	50.8	14.4	49.4	16.0	0.015
	*N*	%	*N*	%	*N*	%	
Gender male	54	75.0	153	75.7	207	75.5	− 899
Iskemia	29	40.3	69	34.2	98	35.8	0.353
Arrhythmia	14	19.4	41	20.3	55	20.1	.877
Respiratory	16	22.2	41	20.3	57	20.8	.730
Hypertension	51	70.8	127	62.9	178	65.0	.225
Diabetes	17	23.6	43	21.3	60	21.9	.682
Oncologic	12	16.7	24	11.9	36	13.1	.304
Renal	7	9.7	21	10.4	28	10.2	.871
Neurologic	13	18.1	31	15.3	44	16.1	.591
Other	18	25.0	70	34.7	88	32.1	.132
Smoking history	9	12.5	17	8.4	26	9.5	<.001
Temperature	61	84.7	184	91.1	245	89.4	.136
Dyspnea	67	93.1	170	84.2	237	86.5	.605
GI	4	5.6	10	5.0	14	5.1	.841
Cough	29	40.3	61	30.2	90	32.8	.119
Myalgia	4	5.6	7	3.5	11	4.0	.442
Astenia	22	30.6	25	12.4	47	17.2	<.001
Headache			1	0.5	1	0.4	.988
Chest pain	1	1.4	5	2.5	6	2.2	.594
Syncope	2	2.8	9	4.5	11	4.0	.538

The majority of cases was represented by males 207 (75.5%), while only 67 (24.5%) were females. The distribution of males and females was not different between groups; males represented 75% in both.

The distribution of comorbidities (cardiovascular diseases, respiratory diseases, arterial hypertension, diabetes, oncological diseases, chronic renal failure, neurological disease, smoking history) was very similar between the two groups of patients. We were not able to collect information about comorbidities in all patients. In the group with fatal events in AAUL-2 was registered a higher percentage of patients with > 3 comorbidities (38 patients, 55.1%) than in the group admitted to the COVID department and ICU (84 patients, 43.1%) (Table 2).

**Table 2:** Number of comorbidities

	AAUL-2 (*N* = 69)	COVID department ICU (*N* = 175)
	*n* (%)	*n* (%)
0	6 (8.69%)	21 (10.77%)
1	10 (14.49%)	39 (20.00%)
2	15 (21.74%)	51 (26.15%)
3 or more	38 (55.07%)	84 (43.08%)

There is evidence that there is a clear difference between the two groups of patients died in AAUL-2 and in the COVID department. A great statistical difference was observed in nearly all physiological and laboratory parameters. The age that is known as an important prognostic factor was considered in the evaluation of the Apache II score. A high difference for Apache II score and GCS that are currently used as index of severity of disease was observed between the two groups.

Patients who were treated in AAUL-2 had a fatal event within 48 h (median 1 day, range 0–2), while patients admitted in the COVID department or in ICU died within 24 days (median 6 days, range 0–24).

The multivariate analysis applied to the physiological and laboratory parameters, the symptoms, and the scores of severity of the disease was able to differentiate patients who died in AAUL-2 and those in the COVID department or in the ICU for the following indicators: asthenia, smoking history, Apache score, GCS, mean arterial pressure, pH, and CRP (Table 3).

**Table 3:** Association with AAUL-2 and COVID department ICU multivariate analysis

Variable	Reference level	Odds ratio	Lower 95%CL	Upper 95%CL	Wald test: *p* value
Asthenia	Yes	3.807	1.418	10.219	0.008
Smoking history	Yes	4.722	1.493	14.935	0.008
Apache score	Continuous	1.166	1.054	1.289	0.003
GCS	Continuous	0.633	0.474	0.847	0.002
PAM	Continuous	0.971	0.945	0.998	0.033
PH	Continuous	< 0.001	< 0.001	0.568	0.035
CRP	Continuous	1.061	1.014	1.110	0.010

## Discussion and Conclusion

This retrospective study reports the experience of the Emergency Department of Humanitas Gavazzeni in Bergamo during the peak of the COVID-19 pandemic.

The epidemiological and clinical characteristics of COVID 19 patients, with the pronominal symptoms and the main comorbidities, were described in the recent paper by Kaur et al [8].

Given the mortality rate of COVID-19, physicians should be aware of the potential risk factors associated with fatal outcome [9–14]. These have been described in the recent literature, however, due to the high rate of mortality observed in our Emergency Department, which is 274 out of 1022 (26.8%); we wanted to focus our attention on our deceased patients in that period (25 February to 26 March 2020). This high rate of death is based both on the severity of this viral-induced disease that progresses rapidly into severe acute respiratory failure and on the tremendous unexpected overload of patients referring to ED. Of course, our structure made its maximum effort to face the situation. Facilities, devices, and personnel were implemented in order to guarantee patients the best assistance as possible. In spite of the lack of validated protocols able to cure these unknown viral infections, physicians optimized the patient’s management in order to provide rational treatments able to control their symptoms. In fact, some of them, in spite of extremely severe conditions, were successfully discharged thanks to the intensive care received [15].

This was done according to the WHO recommendation for endemic, the international literature on COVID-19, and the current indications of the scientific societies [16–19].

We analyzed the most important risk factors able to characterize the dead population, and we observed that some of them resulted very important such as age, oxygen desaturation, pH, CRP, and comorbidities as it was reported by the recent literature [20]. We found also significant differences between 202 patients deceased in COVID department (including ICU) and 72 patients who died in the Acute Admission Unit Level II (AAUL-2). Factors that were able to characterize the difference between these two groups were asthenia, smoking history, Apache score, GCS, mean arterial pressure, pH, and CRP. In particular, the Apache II score (including age for its calculation) was higher in the group died in AAUL-2, while GCS was lower. Patients deceased in AAUL-2 resulted in the worst conditions in comparison with patients who died in the ICU and in COVID-19 department, and their prognostic parameters reflect the situation. All patients received the correct therapy with respect to their clinical status and according to the internal protocols. Patients who were critically ill received palliation and oxygen therapy.

In conclusion this paper reports the most important factors of mortality risk, retrospectively calculated in 1002 patients treated at the ED of Humanitas Gavazzeni Bergamo, in the period 25 February to 26 March 2020 corresponding to the peak of the COVID-19 pandemic in our region. These results were obtained from our experience in a critical situation of emergency and through a monocentric study. They represent a further contribution to the knowledge on the factors that affect the risk of mortality from COVID-19, and in general, they do not differ from the experiences described in similar situations in other parts of the world.
